# The First Recorded *Pasteurella multocida* Infection Post Abdominoplasty Through a Surgical Drain: A Case Report

**DOI:** 10.1093/asjof/ojaf086

**Published:** 2025-07-02

**Authors:** Benyamin A Rozen, Nardin Elias, Noam Armon, Elia Jhonatan, Mor Rittblat

## Abstract

The authors report the first case of postoperative *Pasteurella multocida* infection following elective standard abdominoplasty caused by indirect contamination from a cat-punctured surgical drain. A 51-year-old previously healthy woman underwent uncomplicated standard abdominoplasty but subsequently developed fever, erythema, and abdominal pain. Imaging, aspiration, and microbiological analysis confirmed infection with *P. multocida*. Further questioning revealed her house cat had punctured the surgical drain, which was subsequently repaired by the patient using superglue to restore negative pressure. Targeted antibiotic therapy led to rapid clinical improvement. This case highlights a novel infection transmission pathway and underscores the need for patient education regarding drain management in pet-owning households.

**Level of Evidence: 4 (Therapeutic):**

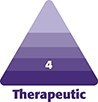

Postoperative infections in aesthetic surgery are most commonly caused by *Staphylococcus aureus* and *Streptococcus* species and are influenced by surgical technique, patient comorbidities, and postoperative care.^1-3^ Although surgical drains are widely utilized to prevent fluid accumulation, their role in reducing surgical-site infections remains uncertain.^4,5^ Although they may help reduce seromas, drains have also been recognized as potential entry points for bacteria; this risk is largely theoretical in the current literature.^4-6^ This concern is mirrored in plastic surgery literature, where Baker et al identified prolonged drain presence as a modifiable risk factor for infection in implant-based breast reconstruction, alongside inconsistent perioperative practices.^6^ At the same time, infections caused by zoonotic pathogens, such as *Pasteurella multocida*, are rare and almost exclusively linked to direct animal bites or scratches.^7-9^

In this report, we present the first case described of indirect postoperative transmission of *P. multocida* through a surgical drain punctured by a household cat. This previously undocumented route of infection highlights a significant gap in postoperative care recommendations and underscores the need for explicit patient education and preventive measures in households with pets.

## CASE PRESENTATION

A 51-year-old woman with no significant medical history and a BMI of 19.8 underwent elective postbariatric standard abdominoplasty with liposuction of the hips without intraoperative complications. Two Jackson–Pratt (JP) drains were placed, and prophylactic cephalexin (1 g orally, 3× daily for 10 days) was initiated. Both drains were removed uneventfully on postoperative day (POD) 10 during routine follow-up.

**Figure 1.**
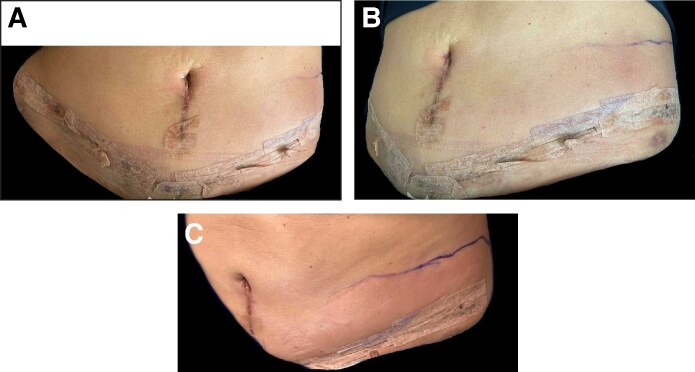
(A-C) A 51-year-old female 12 days status post abdominoplasty. The photographs were taken on presentation to the emergency department. The photographs demonstrate erythema and mild induration localized to the left lower quadrant and lateral waistline. Surgical dressings and drain exit points are visible. These findings preceded the confirmed diagnosis of *Pasteurella multocida* infection.

On POD 12, the patient presented to the emergency department complaining of fever (38.7°C), abdominal wall erythema, and intermittent abdominal pain rated 5/10. Physical examination revealed erythema and mild induration localized to the left lower quadrant of the abdomen and left waistline, without drainage or dehiscence (Figure 1A-C). A computed tomography (CT) scan showed subcutaneous fluid collections suggestive of early loculation in the left abdominal wall. Initial bedside percutaneous aspiration yielded serous fluid. Empirical antibiotics (vancomycin 1.5 g IV single dose and high-dose cefazolin 2 g IV every 8 h for 3 days) were started following infectious disease consultation. She was hospitalized for intravenous therapy and monitoring. Laboratory studies demonstrated leukocytosis (white blood cell [WBC] count 23,000 cells/µL, neutrophils 86%) and elevated C-reactive protein (CRP: 16 mg/L). On POD 13, recurrence of a loculated fluid collection necessitated a second bedside percutaneous drainage, which yielded purulent material containing gram-negative anaerobes. Metronidazole (500 mg orally once daily) was subsequently added.

On POD 14, microbiological testing confirmed *P. multocida* from both aspirate and blood cultures. Upon detailed reassessment, the patient disclosed her cat had punctured 1 JP drain, either with its claws or teeth, prompting her to repair it with superglue to restore negative pressure. The exact mechanism and timing of the puncture could not be determined. Antibiotics were adjusted accordingly to intravenous amoxicillin-clavulanate (1 g/0.2 g every 8 h for 5 days), resulting in significant clinical improvement and notable decline in inflammatory markers (WBC 9700 cells/µL, CRP 9.7 mg/L). She was discharged with an additional 3-week antibiotic course and scheduled for outpatient follow-up.

**Figure 2.**
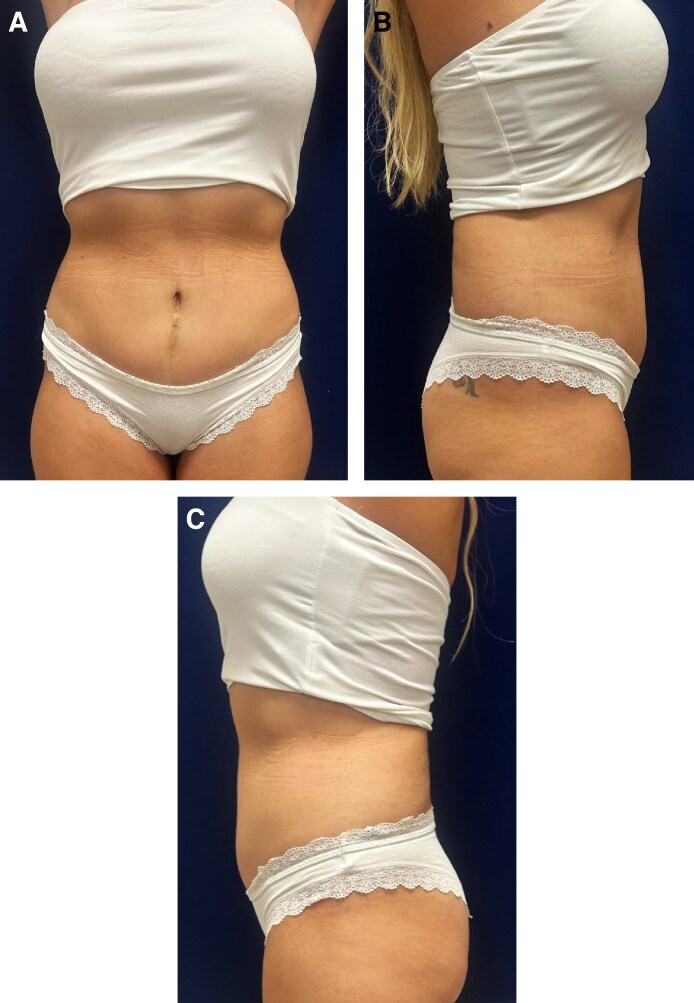
(A-C) A 51-year-old female ∼13 months post abdominoplasty demonstrating complete wound healing without signs of residual infection, scarring complications, or contour irregularities. The patient remained clinically well, with no long-term sequelae.

At her scheduled follow-up visit 1 year postoperatively, the patient demonstrated complete clinical recovery without long-term complications or wound-related sequelae (Figure 2A-C).

## DISCUSSION

Postoperative infections involving *P. multocida*—a gram-negative coccobacillus that colonizes the oropharynx of cats and dogs—typically arise following direct animal bites or scratches.^7-9^ Indirect contamination through surgical drains punctured by household pets appears to be an exceptionally uncommon transmission route for *P. multocida* infection, not only within aesthetic or general surgical literature but also in broader medical contexts.^10-13^ Documented cases of atypical *Pasteurella* transmission have described unusual routes such as inhalation of contaminated secretions or environmental exposure resulting in severe respiratory infections or meningitis; however, these still typically involve some form of direct or close animal contact.^7-9^ For instance, Kannangara et al described a patient who developed severe epiglottitis after consuming food previously eaten by a dog, highlighting indirect transmission through contaminated secretions but without involvement of medical equipment.^8^ Similarly, Chun et al reported a postoperative wound infection resulting from a house cat directly licking a surgical incision following hysterectomy and panniculectomy, but again without any contamination of surgical devices.^9^ The present case, involving contamination of a closed surgical drain punctured by a cat and repaired with superglue by the patient, highlights an unusual, previously undocumented scenario. Although this exposure represents the most plausible source based on the detailed patient history, other less likely environmental or unnoticed pet-related sources of *Pasteurella* contamination cannot be entirely excluded. Nonetheless, this underscores a specific vulnerability that clinicians may overlook, especially when managing postoperative care in pet-owning households.

Interestingly, cephalexin—used here as postoperative prophylaxis—is typically ineffective against *Pasteurella* species.^14^ This scenario, although uncommon, highlights the limited utility of prolonged antibiotic regimens following surgery, irrespective of the presence of drains. Drain removal and meticulous patient instruction on drain management are of utmost importance to prevent such complications.

This case addresses this overlooked route of infection, highlighting a critical area for improved patient safety through clear preoperative and postoperative guidance. Additionally, this case emphasizes the crucial role of thorough and repeated anamnesis; earlier identification of the cat-induced contamination could have expedited targeted antimicrobial therapy and potentially shortened the hospital stay. Considering cats’ inherent curiosity and playful behavior, practical preventive measures—such as explicitly instructing patients on securing drains firmly to their bodies, using protective dressings, or isolating drains from pet access—are strongly recommended. Enhanced clinical awareness and patient education regarding drain care should become routine practice, particularly for pet-owning patients undergoing outpatient aesthetic surgery.

## CONCLUSIONS

This report highlights a previously unpublished indirect transmission route for postoperative infection with *P. multocida* through a cat-punctured surgical drain. Surgeons should proactively incorporate explicit patient education, preventive measures, and meticulous anamnesis into routine preoperative and postoperative guidelines, particularly in pet-owning households. Increased clinical vigilance and early identification of unusual zoonotic exposures are essential to reduce postoperative infection risks and optimize patient safety in aesthetic surgery.
